# Extensive Odontogenic Buccal Space Infection With Multispace Extension in a Diabetic Elderly Patient: A Case Report

**DOI:** 10.7759/cureus.93948

**Published:** 2025-10-06

**Authors:** Sandeep Khandaitkar, Ramakrishna Shenoi, Anant Kahare, Teena Oommen

**Affiliations:** 1 Oral and Maxillofacial Surgery, Ranjeet Deshmukh Dental College and Research Centre, Nagpur, IND

**Keywords:** buccal space infection, diabetes mellitus, infratemporal space, sublingual space, submandibular space

## Abstract

Odontogenic infections are common in oral and maxillofacial practice but may progress rapidly in medically compromised individuals. Diabetes mellitus is a recognized risk factor, predisposing patients to severe and recurrent infections with poor healing potential. We report the case of a 70-year-old diabetic, hypertensive, and hypothyroid female who presented with a left buccal space infection extending to the submandibular, sublingual, and infratemporal spaces, complicated by cortical bone erosion and necrotic lymphadenitis. The patient was managed with incision and drainage, extraction of the offending teeth, and broad-spectrum intravenous antibiotics. This case underscores the aggressive nature of odontogenic infections in diabetic patients and emphasizes the importance of early diagnosis, prompt surgical drainage, systemic control, and multidisciplinary management.

## Introduction

Odontogenic infections arise primarily from pulpal or periodontal pathology and can spread rapidly along fascial planes of the head and neck. While localized infections are usually controlled with drainage and antibiotics, systemic comorbidities such as diabetes mellitus significantly alter the host immune response. Diabetes impairs neutrophil chemotaxis, phagocytosis, and oxidative burst, leading to rapid progression and poor prognosis [1,2].

The most frequently involved fascial spaces include buccal, submandibular, and sublingual regions. However, untreated or aggressive cases may extend to secondary spaces such as infratemporal, parapharyngeal, and mediastinal regions, increasing morbidity and mortality [3,4]. Diabetic patients are especially predisposed because of impaired immunity and chronic low-grade inflammation, which further worsens healing capacity [5].

Chang et al. demonstrated that diabetic patients with odontogenic infections tend to present with more fascial space involvement, higher complication rates, and longer hospital stays compared to non-diabetic patients [6].

## Case presentation

A 70-year-old female presented to the Department of Oral and Maxillofacial Surgery, Ranjeet Deshmukh Dental College and Research Centre, Nagpur, with complaints of swelling on the left side of the face for 8-10 days, gradually increasing in size despite prior oral antibiotics. She reported pain, pus discharge, and restricted mouth opening but denied fever, dysphagia, or voice change.

The patient gave a history of diabetes mellitus (20 years, controlled with oral hypoglycemics), hypertension, and hypothyroidism (controlled). The patient also had a habit of chronic areca nut chewing (30 years). She had been on long-term treatment with metformin 500 mg twice daily for diabetes, amlodipine 5 mg once daily for hypertension, and levothyroxine 75 µg once daily for hypothyroidism. The patient was admitted to the Department of Oral and Maxillofacial Surgery as the primary team. Given her comorbidities, concurrent consultation with the Department of Internal Medicine was obtained to optimize glycemic status and antihypertensive therapy.

Previous outpatient records of glycemic monitoring were available, showing an HbA1c value of 6.6% three months before admission. At the time of admission, laboratory investigations revealed an HbA1c of 6.7%. These findings, in conjunction with the elevated random blood sugar level of 166 mg/dL, suggested that the patient’s diabetes was moderately controlled with ongoing oral hypoglycemic therapy.

**Figure 1:**
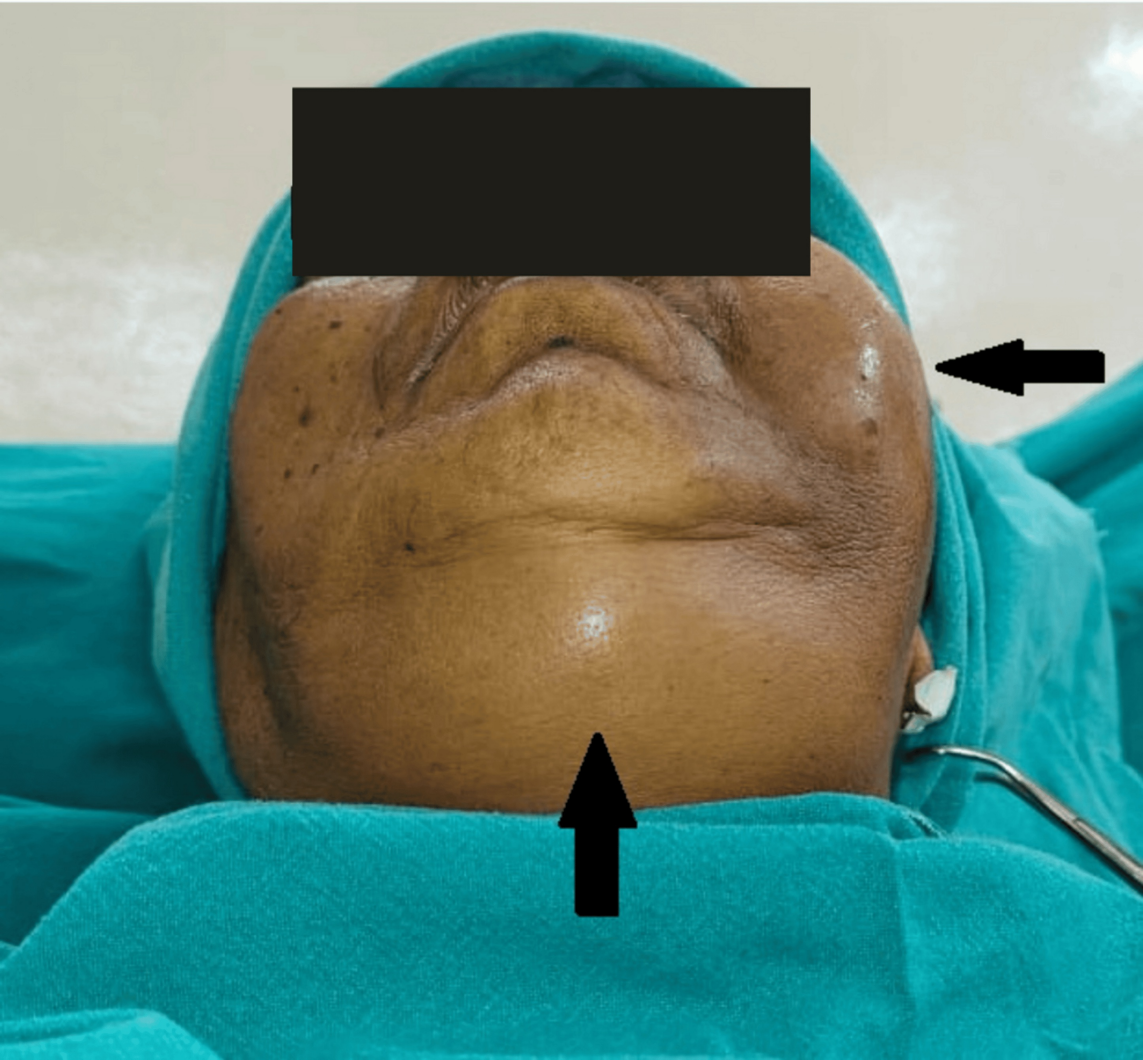
Preoperative extraoral view showing diffuse swelling on the left side of the face and one finger below the lower border of the mandible crossing the midline.

Clinical examination revealed a diffuse extraoral swelling ~11 × 8 × 3 cm with trismus (mouth opening of 1.5 fingers) (Figure 1).

**Figure 2:**
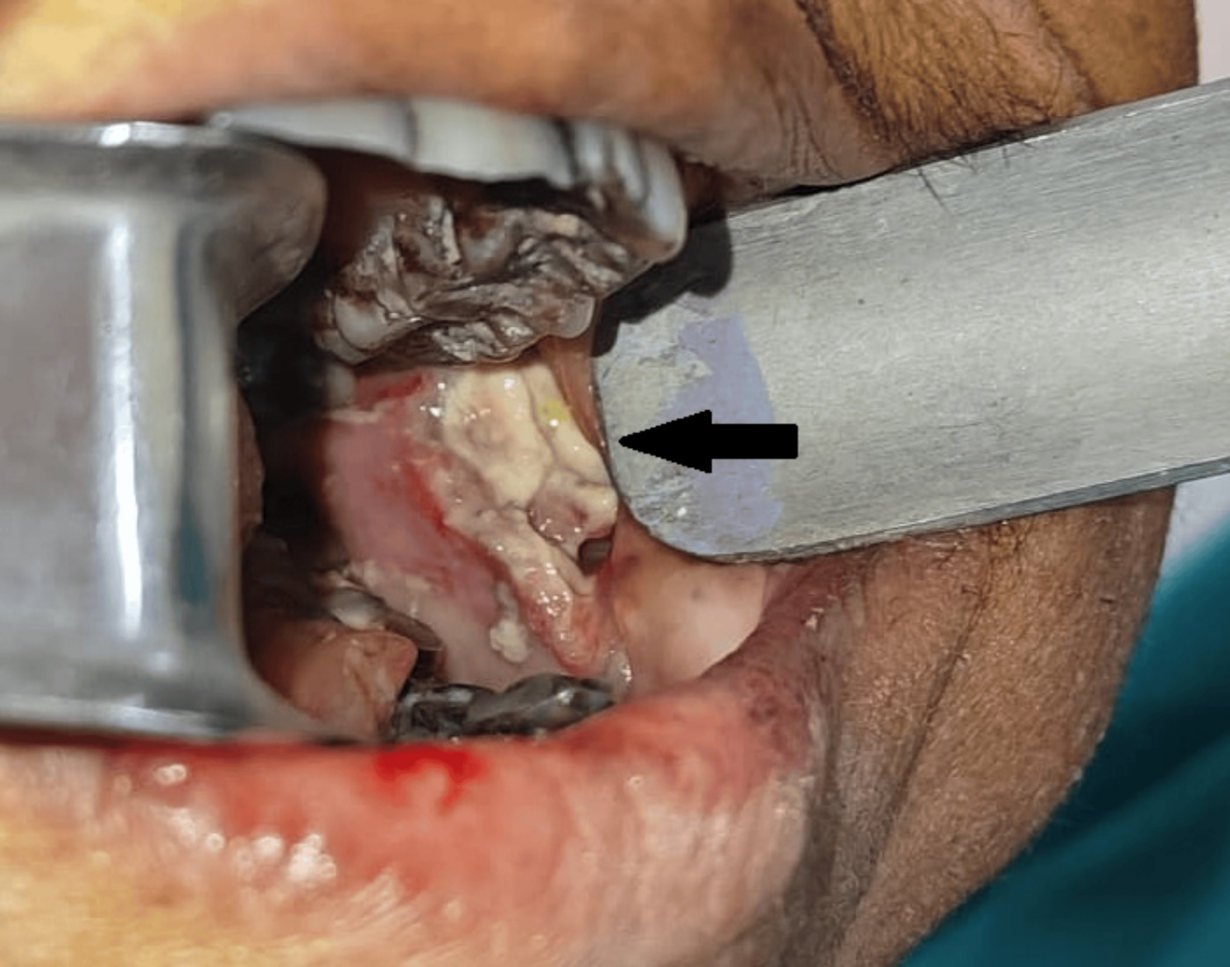
Intraoral view showing necrotic slough and pus discharge from the left buccal mucosa.

Intraoral swelling measured 5 × 4 × 2 cm with whitish slough, pus discharge, and obliteration of the vestibule (Figure 2).

Multiple grossly carious teeth were noted. Laboratory investigations showed anemia, leukocytosis, and hyperglycemia (Table 1).

**Table 1:** Routine laboratory investigations. TLC = total leukocyte count; RBS = random blood sugar; HbA1c = glycated hemoglobin; ALP = alkaline phosphatase; ↓ = decreased; ↑ = increased

Parameter	Patient’s result	Reference range	Unit	Interpretation
Hemoglobin	9.9	12.0–16.0	g/dL	↓ Anemia
TLC	18,000–22,000	4,000–11,000	/mm³	↑ Leukocytosis
RBS	166	70–140	mg/dL	↑ Hyperglycemia
HbA1c	6.7	<5.7 (normal), <7 (good control)	%	Borderline control
ALP	Mildly raised	44–147	IU/L	↑ Mild elevation
Temperature	Afebrile	36.5–37.5	°C	Normal
Blood pressure	Stable	90/60–120/80	mmHg	Normal

**Figure 3:**
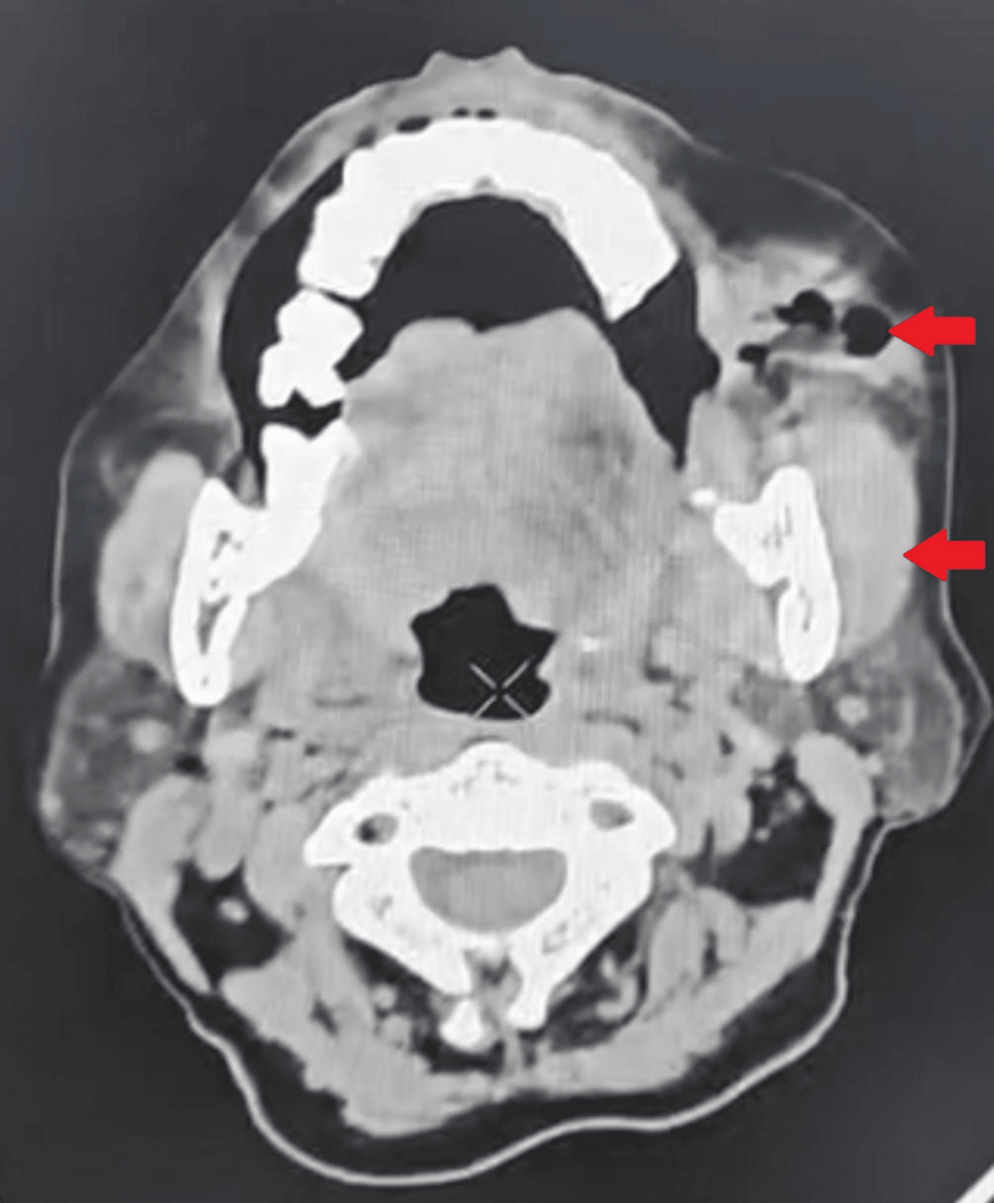
Contrast-enhanced CT revealed multispace involvement (buccal infratemporal).

Contrast-enhanced CT revealed multispace involvement (buccal, submandibular, sublingual, infratemporal) with cortical bone erosion and necrotic lymphadenitis (Figure 3).

**Figure 4:**
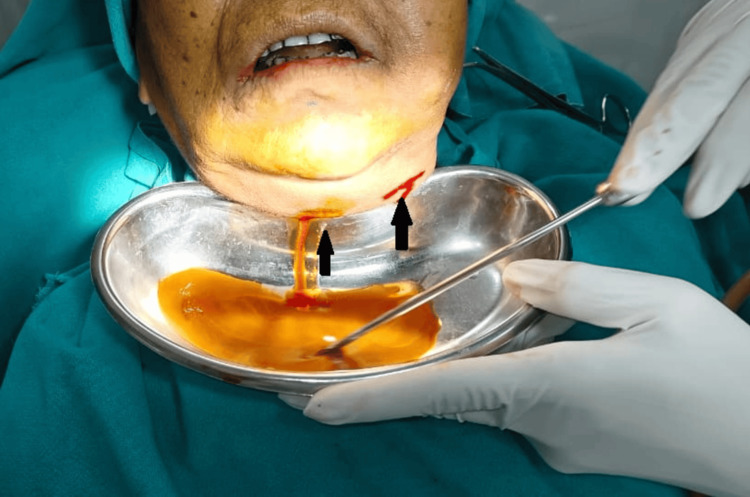
Intraoperative view showing drainage.

Surgical management included intraoral and extraoral incision and drainage, removal of necrotic slough, and debridement of necrotic tissue (Figure 4).

**Figure 5:**
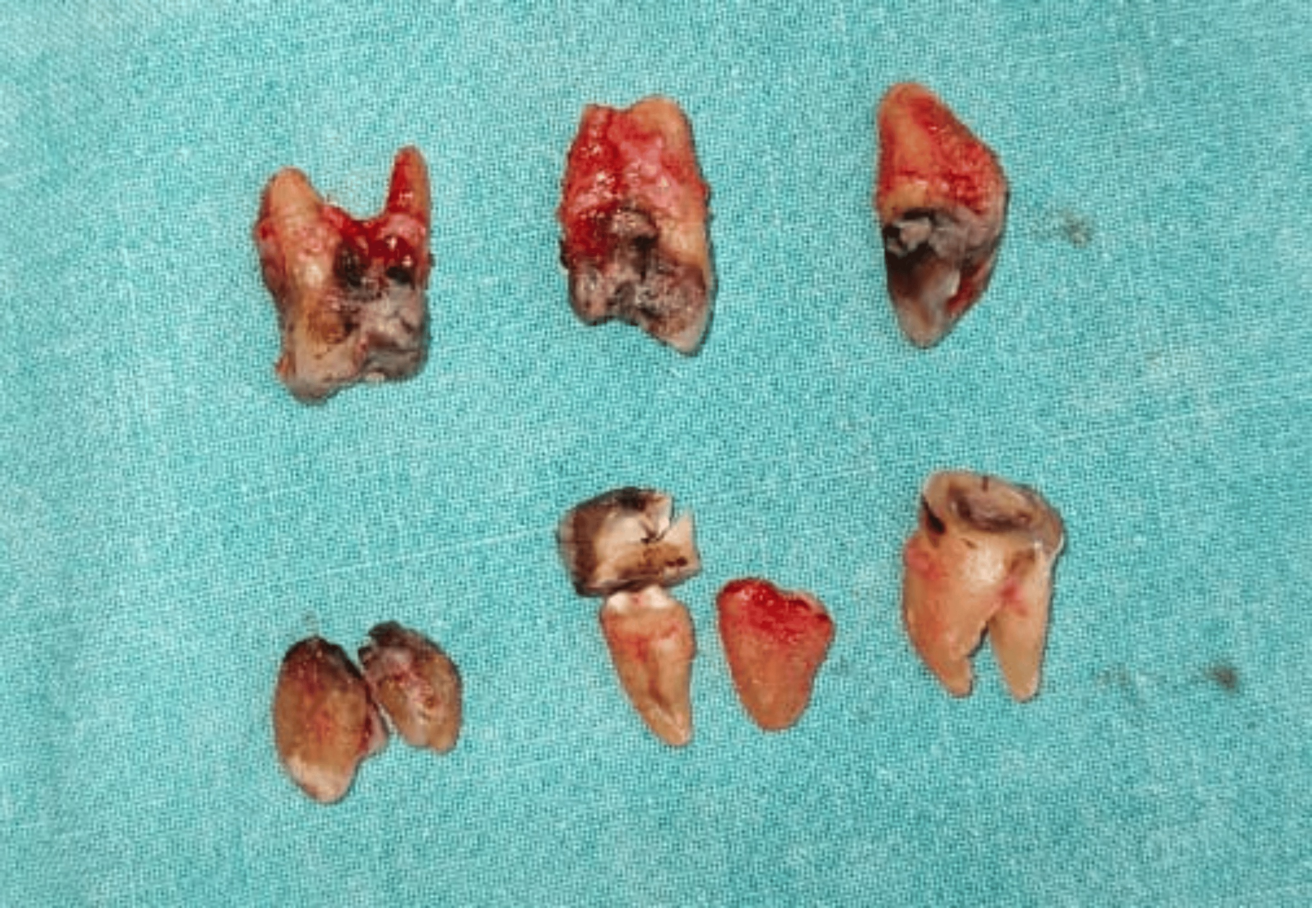
Extracted grossly carious and non-restorable teeth.

Dependent drainage was achieved via a submandibular incision. The offending teeth 21-28 and 31-38 were extracted (Figure 5).

The patient was managed postoperatively with intravenous ceftriaxone and metronidazole, later escalated to piperacillin-tazobactam, along with supportive care and strict glycemic control. She improved gradually and was discharged after 23 days of hospitalization.

## Discussion

Odontogenic infections are among the most common conditions encountered in oral and maxillofacial practice [1]. In immunocompetent individuals, these infections are usually localized, but in immunocompromised patients, particularly those with diabetes mellitus, the course is often aggressive with multispace involvement [2,3].

Diabetes mellitus is associated with altered innate immune responses [4]. Microvascular changes hinder perfusion and delay healing [5]. Chang et al. [6] showed that diabetic patients tend to present with more fascial space involvement, while Huang et al. [7] reported higher complication rates, including necrotizing fasciitis. The buccal space often serves as the initial site before extension to the submandibular and sublingual spaces [8]. Infratemporal spread increases the risk of cavernous sinus thrombosis and airway compromise. If untreated, odontogenic infections may progress to airway obstruction, descending necrotizing mediastinitis, or septicemia [9,10], with mortality especially high in diabetics [11].

Our patient demonstrated rapid progression of a buccal space infection into submandibular, sublingual, and infratemporal spaces, with radiological evidence of cortical erosion and necrotic lymphadenitis. The differential diagnoses considered included suppurative odontogenic cellulitis and deep neck infections of non-odontogenic origin, such as salivary gland suppuration or tuberculous lymphadenitis. Odontogenic cellulitis was excluded as imaging revealed multiple loculated abscesses with cortical bone erosion rather than diffuse soft tissue swelling. Non-odontogenic deep neck infections were also ruled out, as the infection was clearly contiguous with grossly carious teeth and responded well to extraction and surgical drainage.

Management principles include elimination of the source, surgical drainage, and systemic antibiotic therapy [1,2]. In this case, multiple decayed teeth were extracted, drainage was established, and intravenous antibiotics were escalated according to clinical response [12,13]. Supportive measures, particularly strict glycemic control, were critical. Adjuncts such as negative-pressure wound therapy and culture-guided antibiotics improve prognosis [14].

This case is significant because it demonstrates uncommon severity, emphasizes the need for vigilance in diabetics, highlights educational value for clinicians, and underlines global importance given rising diabetes prevalence [15,16].

## Conclusions

Odontogenic infections in elderly diabetic patients can progress rapidly with multispace extension and bone destruction. Early recognition, prompt surgical drainage, removal of the odontogenic foci, and broad-spectrum intravenous antibiotics, alongside strict systemic control, are key. This case demonstrates how a common dental infection can evolve into a severe, life-threatening condition in the presence of diabetes mellitus.
